# Chromatin Changes in Dicer-Deficient Mouse Embryonic Stem Cells in Response to Retinoic Acid Induced Differentiation

**DOI:** 10.1371/journal.pone.0074556

**Published:** 2013-09-09

**Authors:** Jayantha B. Tennakoon, Hongran Wang, Cristian Coarfa, Austin J. Cooney, Preethi H. Gunaratne

**Affiliations:** 1 Department of Biology and Biochemistry, University of Houston, Houston, Texas, United States of America; 2 Department of Molecular and Cellular Biology, Baylor College of Medicine, Houston, Texas, United States of America; 3 Department of Pathology, Baylor College of Medicine, Houston, Texas, United States of America; 4 Human Genome Sequencing Center, Baylor College of Medicine, Houston, Texas, United States of America; University of Minnesota Medical School, United States of America

## Abstract

Loss of *Dicer*, an enzyme critical for microRNA biogenesis, results in lethality due to a block in mouse embryonic stem cell (mES) differentiation. Using ChIP-Seq we found increased H3K9me2 at over 900 CpG islands in the Dicer^-/-^ES epigenome. Gene ontology analysis revealed that promoters of chromatin regulators to be among the most impacted by increased CpG island H3K9me2 in ES (Dicer^-/-^). We therefore, extended the study to include H3K4me3 and H3K27me3 marks for selected genes. We found that the ES (Dicer^-/-^) mutant epigenome was characterized by a shift in the overall balance between transcriptionally favorable (H3K4me3) and unfavorable (H3K27me3) marks at key genes regulating ES cell differentiation. Pluripotency genes *Oct4*, *Sox2* and *Nanog* were not impacted in relation to patterns of H3K27me3 and H3K4me3 and showed no changes in the rates of transcript down-regulation in response to RA. The most striking changes were observed in regards to genes regulating differentiation and the transition from self-renewal to differentiation. An increase in H3K4me3 at the promoter of *Lin28b* was associated with the down-regulation of this gene at a lower rate in Dicer^-/-^ES as compared to wild type ES. An increase in H3K27me3 in the promoters of differentiation genes *Hoxa1* and *Cdx2* in Dicer^-/-^ES cells was coincident with an inability to up-regulate these genes at the same rate as ES upon retinoic acid (RA)-induced differentiation. We found that siRNAs *Ezh2* and post-transcriptional silencing of *Ezh2* by let-7g rescued this effect suggesting that *Ezh2* up-regulation is in part responsible for increased H3K27me3 and decreased rates of up-regulation of differentiation genes in Dicer^-/-^ES.

## Introduction

*Dicer* is an RNAse III type endoribonuclease with specific enzymatic activity that cleaves double stranded RNA molecules [1]. *Dicer*-dependent microRNAs (miRNAs) are initiated through RNA polymerase II activity to generate a primary miRNA that forms a stem and loop structure which is cleaved by the combined activities of two enzymes of the microprocessor complex, Drosha and DGCR8/Pasha [2]. The resultant stem loop precursors are then transported to the cytoplasm through Exportin-5 action where *Dicer* catalyzes the second cleavage event to produce 18 to 25 nucleotide mature miRNA duplex structures [2]. Mature miRNAs are known to regulate gene expression by either cleaving mRNA molecules by binding to respective 3’ UTR elements or by translational repression [2–4]. The conventional role of miRNAs in posttranscriptional gene silencing is well established, however the full extent of the role of *Dicer*-dependent pathways beyond their established role in regulatory mechanisms such as epigenetic silencing critical for biological processes has yet to be determined. In spite of several studies having shown small RNA molecules regulating epigenetic modifications in plants, flies and fission yeast [5–7] it remains unclear whether similar regulation occurs in higher order mammals.

Studies have shown that *Dicer* deficient mouse embryos show morphological abnormalities at day 6.5 of embryonic development [8–10]. Critical networks that *Dicer*-dependent miRNAs regulate in maintaining mammalian stem cell pluripotency and cellular transition by functioning as repressors of transcription factors, chromatin modifiers and cell signaling molecules was reported by Marson et al. in 2008 [11]. Moreover it is known that miRNAs are needed for proper maintenance of DNA methylation in mES cells [12]. The currently proposed mechanistic principle is that DNA methylation is invoked in ES cells by down regulation of repressors of DNA methyltransferases 3A and 3B (Dnmt3) such as Rbl in part by miRNAs in the miR-290-295 cluster ensuring availability of DNA methyltransferases for *de novo* DNA methylation [12]. The critical roles which histone modifications play in determining cellular differentiation and stem cell plasticity is all the more emphasized through observations made by Boyer and co-workers [13] that polycomb group proteins repress key developmental regulators in mES cells through repressive histone H3K27me3 modifications in the pluripotent state. Bernstein et al. 2006 reported that the co-occupation of key developmental regulators by transcriptionally unfavorable H3K27me3 as well as transcriptionally favorable H3K4me3 modifications render these subsets of genes to be repressed during the pluripotent state poised for activation upon differentiation [14].

We hypothesized that *Dicer*-dependent pathways, either directly or indirectly through miRNA mediated regulation, guide epigenetic changes in mES cells and affect transcript levels of genes that are critical for stem cell function. To address our hypothesis we mapped genome-wide changes in H3K9me2 using ChIP-Seq analysis of WT ES cells and Dicer^-/-^ES cells. We found a significant increase in H3K9me2 at over 900 CpG islands in Dicer^-/-^ES. Gene ontology analysis indicated that CpG islands of chromatin modifiers were significantly impacted. Therefore, we extended this work to study additional histone marks including H3K27me3 and H3K4me3 in the promoter regions of genes critical for the maintenance and differentiation of ES cells. H3K9me2 and H3K27me3 are considered context dependent repressive marks while H3K4me3, which is favorably linked with polymerase recruitment is typically associated with transcriptional activation. Focusing on key genes involved mES self-renewal and pluripotency (*Oct4*, *Nanog*, *Sox2* and *Ronin*), differentiation (*Cdx2* and *Hoxa1*) and genes regulating the transition between these states (*Lin28*b and *Gcnf*) we systematically measured levels of transcripts and associated changes in the above mentioned histone modifications in Dicer^-/-^ ES as compared to WT ES [15–17]. . 

Taken together our data showed that loss of *Dicer* impacts the balance between transcriptionally favorable and unfavorable histone modifications affecting the rates of changes and expression levels of mRNA of key developmental genes. miRNA mediated rescue experiments, which we subsequently carried out showed that changes in chromatin state, and mRNA expression associated with loss of *Dicer* is reversible by targeted silencing of *Ezh2*. In summary, our data show that *Dicer* directly or indirectly regulates gene expression in mES cells at the epigenetic as well as post-transcriptional levels and point to a critical role *Dicer* plays in mammalian embryogenesis.

## Results

### a: Loss of Dicer has a strong impact on H3K9me2 in the epigenomic landscape in mES cells

**Figure 1:**
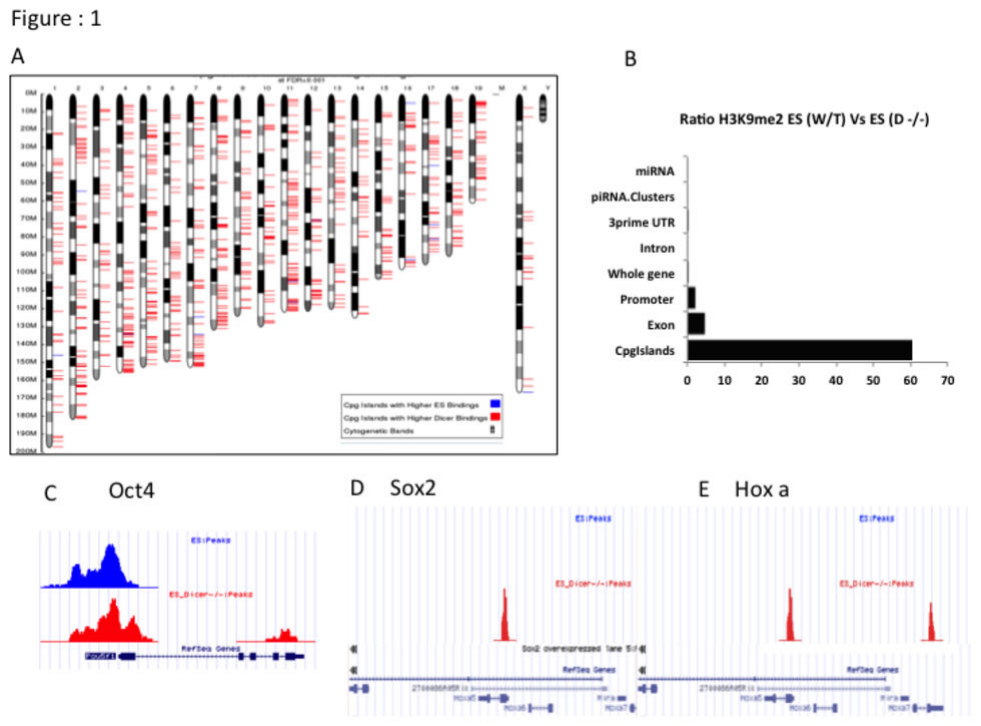
*Dicer*’s effect on H3K9me2 distribution patterns in mES cells. A) Differential distribution of H3K9me2 in mouse chromosomes. Blue bars represent loci where higher enrichment levels were observed in mES cells while red bars represent loci where enrichment levels were higher in Dicer^-/-^ ES cells. Specific genomic loci can be found in table **S3**. B) Bar graph showing ratio of H3K9me2 occupation in Dicer^-/-^ ES cells compared to WT ES cells in different genomic elements. C, D and E) Sequence tags from H3K9me2 ChIP-Seq experiment mapped to the UCSC genome browser to show enrichment at *Oct4* and *Sox2* and *Hoxa* gene cluster promoter regions of WT ES and Dicer^-/-^ ES cells. Blue peaks represent WT ES cells and red peaks represent Dicer^-/-^ ES cells.

It has been shown that small RNAs regulate epigenetic changes in plants, flies and fission yeast [5–7]. However a potential role for *Dicer*-dependent pathways and miRNAs in mammals in particular remain hitherto unexplored. It has also been shown that miRNAs regulate DNA methylation via the miR-290-295 candidates functioning as repressors of Rbl2, which in turn is a repressor of Dnmt3 [12]. H3K9me2 modifications are widely considered epigenetic marks that demarcate genetic regions bound to undergo DNA methylation [18,19]. In order to assess *Dicer*’s effect on potential upstream events regulated via H3K9me2 modifications we carried out genome-wide H3K9me2 ChIP-Seq analysis using the Illumina platform. We then generated genome-wide H3K9me2 maps on the mES cell genome using the PASH algorithm [20]. Of the regions showing differential H3K9me2 modification we found increased H3K9me2 in Dicer^-/-^ ES cells in 71% of promoter regions and 98% of CpG islands, (Figure 1A). Comparison of ratios of H3K9me2 occupation by the number of tags mapping to different genomic features revealed that on a global scale Dicer^-/-^ ES cells showed fold changes of 60 at CpG islands, 5 at exons and 2 at promoters and <1 in whole genes, introns, 3’ UTR whole gene, 3’ UTR piRNA and miRNA clusters respectively (Figure 1B). These observations strongly suggested either a direct or indirect role for *Dicer*-dependent pathways in regulating the mES cell epigenome via H3K9me2 modifications. In order to see if the biological processes that are impacted by the large number of genes associated with increased CpG island H3K9me2 and the small number of genes associated with decreased CpG island H3K9me2 in Dicer^-/-^ ES are distinct we carried out GO analyses on the gene sets. From data shown in table 1 we see that genes with equivalent levels of CpG island H3K9me2 in WT and Dicer^-/-^ ES and genes exhibiting higher levels of CpG island H3K9me2 in WT were associated with housekeeping functions such as nucleosome and cellular component assembly, amino acid biosynthesis, protein acetylation and cation transport. In sharp contrast genes associated with higher levels of CpG island H3K9me2 in Dicer^-/-^ ES cells included chromatin regulators, genes regulating DNA damage and repair and cell cycle. The same was true when we examined key genes involved in ES cell self-renewal and differentiation. Pluripotency genes such as *Oct4* and *Nanog* showed equivalent levels of H3K9me2 in WT and Dicer^-/-^ ES (Figure 1C). In contrast, genes in the *Hox* cluster that are induced during ES cell differentiation showed increased CpG island H3K9me2 in Dicer^-/-^ ES (Figure 1E). To more closely assess the effects of loss of *Dicer* on promoter H3K9me2 enrichment levels of key developmental genes we carried out ChIP-qPCR assays and found statistically insignificant subtle differences in H3K9me2 levels between the two cell lines subject to RA induced differentiation Figure S1 (A to G).

**Table 1:** Gene ontology terms of differentially methylated CpG island H3K9me2 sites.

***Dicer* Specific**	**ES Specific**	**Common ES-*Dicer***
Chromatin regulator	BP00014:Amino acid biosynthesis	GO:0000786~nucleosome
DNA damage	BP00066:Protein acetylation	GO:0006334~nucleosome assembly
DNA repair	BP00143:Cation transport	GO:0022607~cellular component assembly
GO:0000166~nucleotide binding	BP00201:Skeletal development	GO:0022829~wide pore channel activity
GO:0000785~chromatin	BP00204:Cytokinesis	GO:0065003~macromolecular complex assembly
GO:0003676~nucleic acid binding	BP00289:Other metabolism	MF00101:Guanyl-nucleotide exchange factor phosphoprotein
GO:0003677~DNA binding		GO:0048471~perinuclear region of cytoplasm
GO:0004672~protein kinase activity		MF00060:Damaged DNA-binding protein
GO:0005488~binding		MF00178:Extracellular matrix Nucleosome core
GO:0005515~protein binding		
GO:0005524~ATP binding		
GO:0005622~intracellular		
GO:0005634~nucleus		
GO:0005694~chromosome		
GO:0005737~cytoplasm		
GO:0005921~gap junction		
GO:0006139~nucleobase, nucleoside, nucleotide and nucleic acid metabolic process		
GO:0006259~DNA metabolic process		
GO:0006323~DNA packaging		
GO:0006325~establishment and/or maintenance of chromatin architecture		
GO:0006333~chromatin assembly or disassembly		
GO:0006464~protein modification process		
GO:0006468~protein amino acid phosphorylation		
GO:0006793~phosphorus metabolic process		
GO:0006796~phosphate metabolic process		
GO:0007049~cell cycle		
GO:0007242~intracellular signaling cascade		
GO:0008152~metabolic process		
GO:0008270~zinc ion binding		
GO:0009987~cellular process		
GO:0016310~phosphorylation		
GO:0016773~phosphotransferase activity, alcohol group as acceptor		
GO:0017076~purine nucleotide binding		
GO:0022403~cell cycle phase		

### b. H3K27me3 modifying *Ezh2* is overexpressed in Dicer^-/-^ ES cells whereas H3K4me3/H3K36me3/H3K9me2 modifying enzyme gene expression levels are comparable in both cell lines.

**Figure 2:**
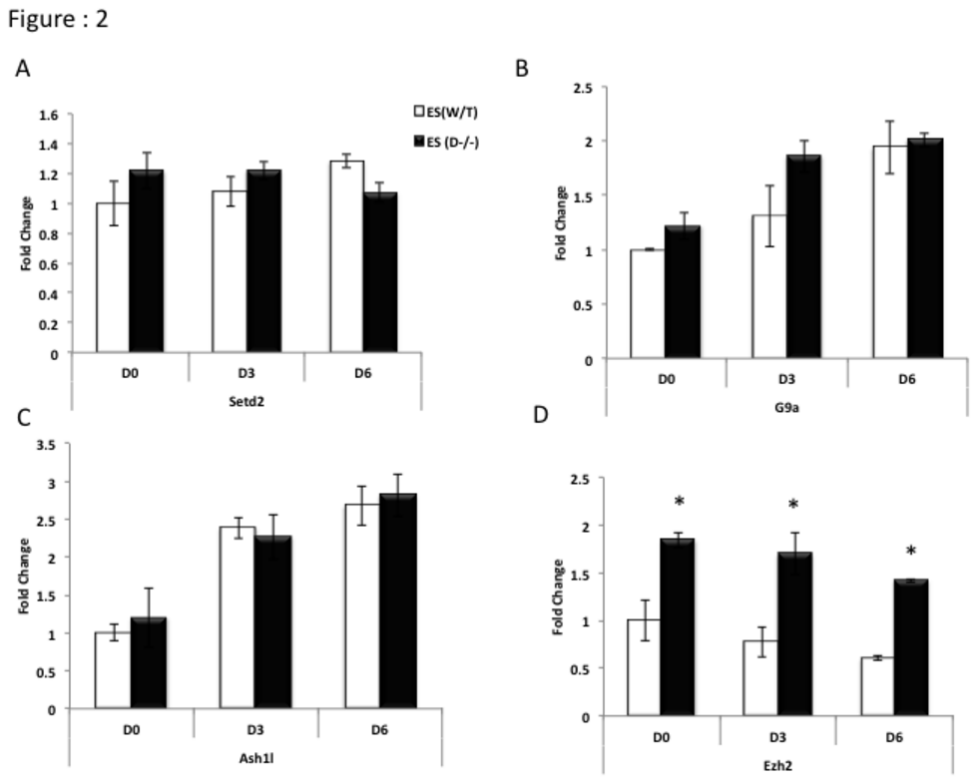
*Ezh2* transcripts in Dicer^-/-^ ES cells are overexpressed compared to WT ES cells, while *Setd2*, *G9a* and *Ash1l* levels remain relatively similar in both cell lines. A, B C and D bar graphs showing expression levels of the histone methyl transferases *Setd2*, *G9a*, *Ash1l* and *Ezh2* in WT and Dicer^-/-^ ES cells upon RA treatment through days 0 (D0) to day 6 (D6). Note that the transcripts of enzymes *Setd2* and *Ash1l*, which promote H3K36me3 and H3K4me3 modifications respectively show comparable results while H3K9me2 promoting *G9a* is slightly elevated at a statistically insignificant level in Dicer^-/-^ ES cells and *Ezh2* is significantly high in Dicer^-/-^ ES cells. The symbol* indicates that the results of a given time point were significantly different between the two cell lines at a confidence level of 0.05 when a students t-test was performed.

To ascertain how loss of *Dicer* affects the transcript levels of histone modifying and DNA modifying enzymes, which contribute downstream to elevated or reduced levels of corresponding epigenetic modifications we carried out qRT-PCR analysis on a panel of epigenetic modifier genes in WT and Dicer^-/-^ ES cells. While we found no significant differences in H3K4me3, and H3K36me3 modifying enzymes *Setd2* and *Ash1l* gene transcript levels (Figure 2A and 2C) we found slightly elevated statistically insignificant levels of the H3K9me2 modifying enzyme *G9a* transcripts in Dicer^-/-^ ES cells (Figure 2B). Most notably the transcript levels of H3K27me3 modifying enzyme *Ezh2* [21] was consistently overexpressed in Dicer^-/-^ ES cells compared to WT cells from day 0 through day 6 of RA induced differentiation (Figure 2D).

### c: Pluripotency genes Oct4, Sox2 and Nanog are not impacted by loss of Dicer but Ronin levels are significantly lower in Dicer^-/-^ ES cells

**Figure 3:**
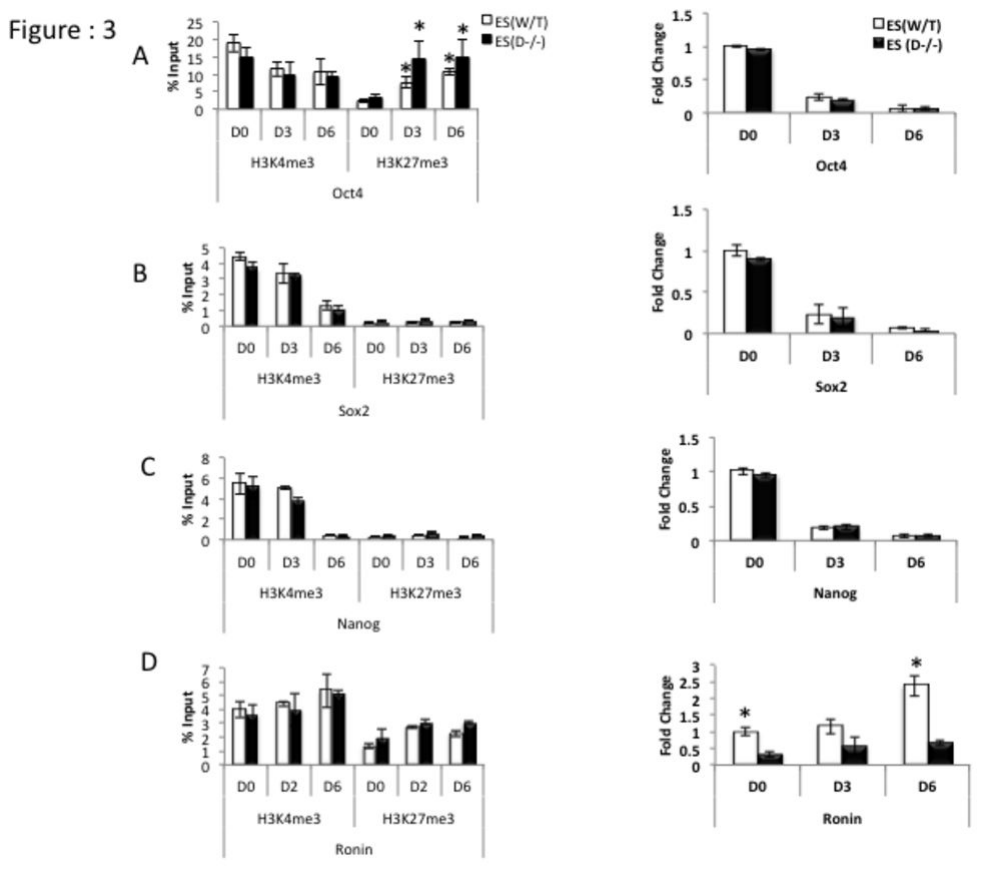
Enrichment levels of transcriptionally favorable versus transcriptionally unfavorable histone modifications in pluripotency factor gene promoter regions and their mRNA expression in mES cells. A, B, C and D left ChIP-qPCR and right panels qRT-PCR results of relative mRNA levels in *Oct4*, *Sox 2*, *Nanog* and *Ronin* in WT and Dicer^-/-^ES cells upon RA induced differentiation through days 0 (D0) to 6 (D6). A and B right panels transcripts of *Oct4* and *Sox2* go down both in mES and Dicer^-/-^ES cells upon RA treatment (A and B left panels). Transcriptionally favorable (H3K4me3) and unfavorable (H3K27me3) presence at *Oct4* and *Sox2* promoters in WT ES and Dicer^-/-^ ES (C right panel). Expression levels of *Nanog* go down upon RA induction while transcriptionally favorable H3K4me3 marks gradually go down in both cell lines (C left panel) (D left panel). H3K4me3 and H3K27me3 occupation at the Ronin promoter (D right panel). Transcripts of *Ronin* go up at a higher rate in Dicer^-/-^ ES cells compared to WT ES cells. Figure 3A Left panel * indicates that the difference for a given cell line at a given time point was significant when compared to day zero of the same cell line at 0.05 confidence levels when a students t-test was performed. Figure 3D right panel * indicates that the difference at a given time point between the two cell lines were significant at 0.05 confidence levels when a students t-test was performed.

*Dicer*-deficient embryos tend to show morphological abnormalities as early as day 6.5 of development [8,9]. It has also been shown that *Dicer*-deficient ES cells are defective in their ability to properly differentiate [9]. In order to assess the degree to which *Dicer*-deficient ES cells differentiate and to uncover mechanisms through which *Dicer* potentially regulates cellular transition we carried out ChIP-qPCR assays in the promoter regions of key genes regulating self-renewal and differentiation for transcriptionally favorable H3K4me3 versus transcriptionally unfavorable H3K27me3 marks side by side with qRT-PCR assays to determine gene expression. We particularly chose the H3K27me3 mark because *Ezh2* mRNA levels were high in Dicer^-/-^ ES cells and H3K4me3 mark as it is associated with active polymerase recruitment. Upon RA induction transcripts of pluripotency factors *Oct4 Sox2* and *Nanog* gradually went down in both WT and Dicer^-/-^ ES cells at comparable rates (Figure 3A–C right panels). However transcripts of *Ronin* (Figure 3D right panel) was found to be significantly lower in Dicer^-/-^ ES cells. ChIP-qPCR on promoter regions of the pluripotency genes *Oct4, Sox2*, *Nanog and Ronin* showed no consistent change in H3K4me3 and H3K27me3 marks in Dicer^-/-^ ES cells compared to WT ES cells (Figure 3A to 3D left panels). However, an increase of H3K27me3 marks in response to RA treatment was noticeable in both cell lines at the *Oct4* promoter (Figure 3A Left panel).

### d: Transcriptionally favorable histone modifications in promoter regions impede the down-regulation of *Lin28*b in Dicer^-/-^ ES

**Figure 4:**
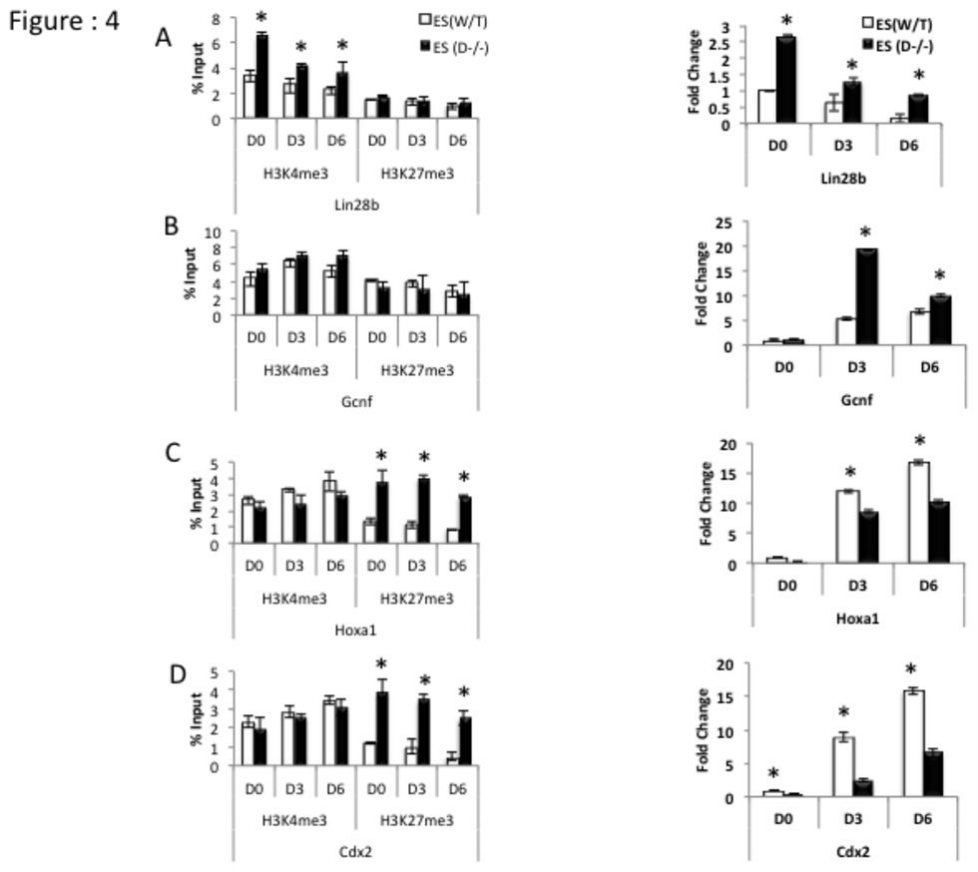
Transcriptionally favorable H3K4me3 modifications in promoter regions increase expression levels of *Lin28*b in Dicer^-/-^ ES cells relative to WT ES cells, while H3K27me3 histone modifications disfavor *Hoxa1* and *Cdx2* expression in Dicer^-/-^ ES cells relative to WT ES. AB C and D left panels, ChIP- qPCR results on promoter regions and qRT-PCR results (Right panles) of *Lin28*b, *Gcnf*, *Hoxa1* and *Cdx2* respectively upon RA induction from day 0 (D0) through day 6 (D6). Note higher enrichment levels of H3K4me3 in the promoter regions of *Lin28*b (A left panel) which affect increased expression levels in Dicer^-/-^ ES cells (A right panel). As shown in C and D left panels higher occupation of H3K27me3 in *Hox a1* and *Cdx2* attenuates upregulation of transcripts in Dicer^-/-^ ES cells compared to WT ES cells (C and D right panels). The symbol * indicates that the difference at a given time point between the two cell lines were significant at 0.05 confidence levels when a students t-test was performed.

*Lin28*b and *Gcnf* are genes known to facilitate the transition from ES self-renewal to differentiation. *Lin28*b decreases and *Gcnf* increases upon RA-induced differentiation [22–24]. We examined both; epigenetic changes in promoter regions and transcript levels of these genes in Dicer^-/-^ ES cells treated with RA and compared this with WT. In Dicer^-/-^ ES cells the promoter region of *Lin28*b exhibited high enrichment of H3K4me3 compared to ES cells while the *Gcnf* promoter region showed slightly elevated yet statistically insignificant levels of enrichment (Figure 4A and B left panels). At the transcript level *Lin28*b failed to go down at the same rate in Dicer^-/-^ ES cells upon RA induction as compared to WT (Figure 4A right panel). It is possible that the presence of higher levels of H3K4me3 marks at their promoter regions favoring transcription in Dicer^-/-^ ES cells is in part responsible for this. *Gcnf* as well was observed to go up at a faster rate in Dicer^-/-^ ES cells upon RA induction as compared to WT (Figure 4B right panel).

### e: Transcriptionally unfavorable histone modifications impede the up-regulation of Hoxa1 and Cdx2 during RA-induced differentiation in Dicer^-/-^ ES cells

In order to examine the impact of *Dicer*-dependent histone modifications on genes promoting ES cell differentiation we carried out ChIP-qPCR and qRT-PCR analysis on *Cdx2* and *Hoxa1*. Here we noted an entirely different outcome to the other genes analyzed so far. Dicer^-/-^ ES cells retained the ability to induce transcripts upon RA-treatment but failed to do so at the same rate as WT cells (Figure 4C and 4D right panels). In addition the promoter regions of *Cdx2* and *Hoxa1* had significantly higher levels of H3K27me3 levels in promoter regions in Dicer^-/-^ ES cells compared to WT cells, which likely explains the attenuated up-regulation of these genes upon RA-induction of Dicer^-/-^ ES cells. Comparable and high H3K4me3 marks were found in both cell lines (Figure 4C and 4D left panels).

### f: Reduced transcript levels of Hoxa1 and Cdx2 in Dicer^-/-^ ES cells can be rescued by overexpressing let-7g miRNA or siRNAs targeting Ezh2

Based on 1) the failure of *Hoxa1* and *Cdx2* to be fully transcriptionally induced upon RA treatment in Dicer^-/-^ ES cells, 2) increased enrichment of H3K27me3 in the promoter regions of *Hoxa1* and *Cdx2* in Dicer^-/-^ ES cells, and 3) significant increase in transcript levels of *Ezh2* that has been established to regulate H3K27me3 in WT ES cells we hypothesized that a key developmentally regulated miRNA lacking in Dicer^-/-^ ES cells due to miRNA biogenesis defects may play a mechanistic role in facilitating higher levels of *Ezh2* transcripts and hence higher enrichment of H3K27me3 marks in *Hoxa1* and *Cdx2* in promoter regions. Moreover *Lin28*b, which is known to play a critical role in facilitating cell fate through a double negative feed back loop between let-7g and itself [23], was found to be substantially increased in Dicer^-/-^ ES cells and let-7g is predicted to target *Ezh2*. From these observations we hypothesized that the loss of let-7g in Dicer^-/-^ ES cells led to the increase in its predicted target *Ezh2*, which in turn led to increased H3K27me3 at the *Hoxa1* and *Cdx2* promoter regions. It is possible that failure to fully induce genes that are critical for embryonic differentiation such as *Hoxa1* and *Cdx2* are a result of the failure to down-regulate epigenetic modifiers such as *Ezh2* which potentially play an important role in the embryonic lethality of the Dicer^-/-^ embryos.

**Figure 5:**
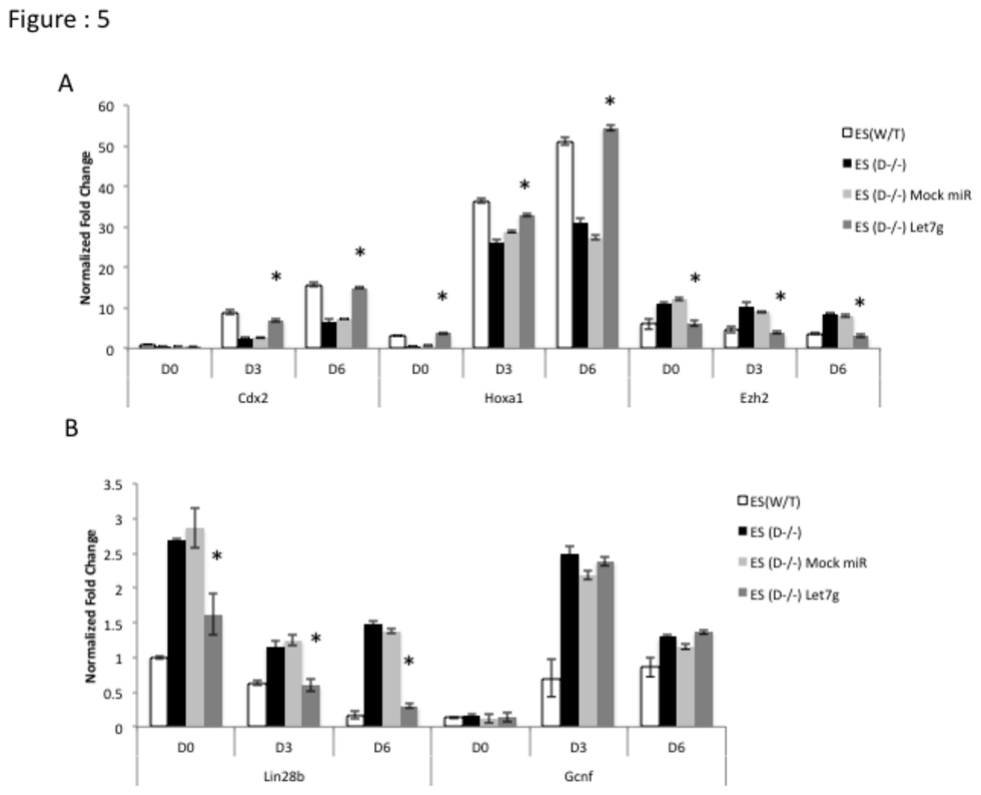
Transfecting Dicer^-/-^ ES cells with let-7g miRNA rescues *Hoxa1* and *Cdx2* transcript levels during retinoic acid induced differentiation. A) *Hoxa1* and *Cdx2* transcript levels increase upon transfecting Dicer^-/-^ ES cells with miRNA let-7g while *Ezh2* transcript levels go down. B) *Lin28*b transcripts are significantly reduced in Dicer^-/-^ES cells upon transfecting with miRNA let-7g. * indicates that the difference at a given time point between Dicer^-/-^ ES cells and Dicer^-/-^ ES cells with let-7g were significant at 0.05 confidence levels when a students t-test was performed.

**Figure 6:**
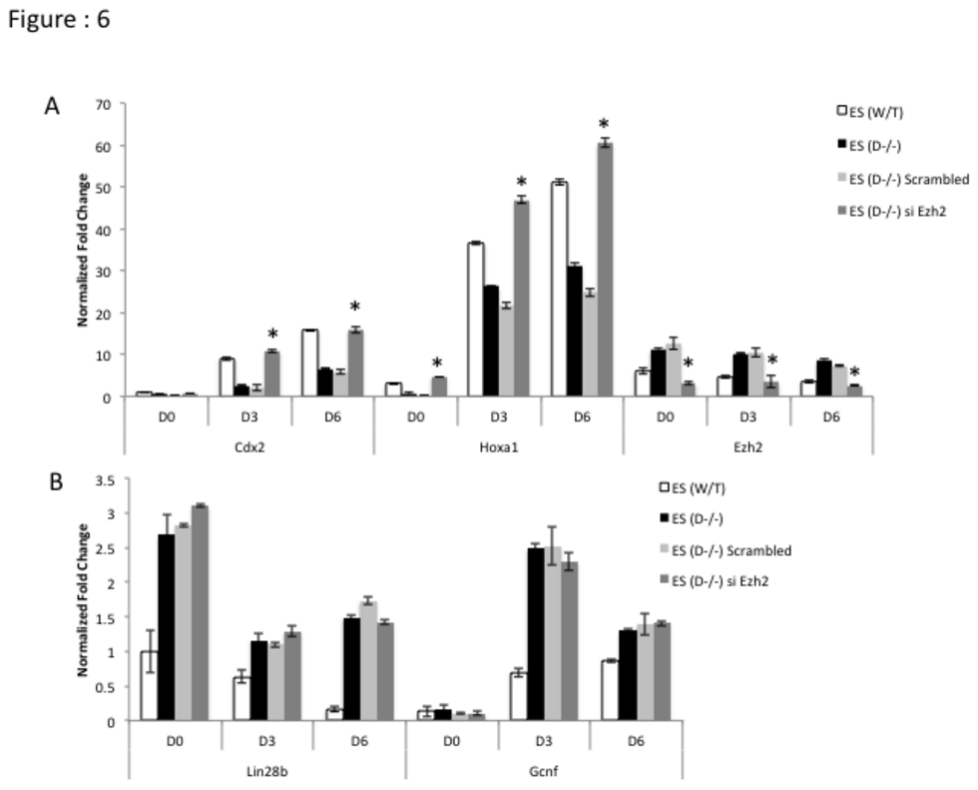
Transfecting Dicer^-/-^ ES cells with siRNAs specifically targeting *Ezh2* rescues *Hoxa1* and *Cdx2* transcript levels during retinoic acid induced differentiation. A) *Ezh2* transcript levels reduce while *Hoxa1* and *Cdx2* transcript levels increase upon transfecting Dicer^-/-^ ES cells with siRNA specific to *Ezh2*. B) *Lin28*b transcripts remain significantly unchanged upon transfecting Dicer^-/-^ES cells with siRNA specifically targeting *Ezh2*. * Indicates that the differences at a given time point between Dicer^-/-^ ES cells and Dicer^-/-^ ES cells with si*Ezh2* were significant at 0.05 confidence levels when a students t-test was performed.

**Figure 7:**
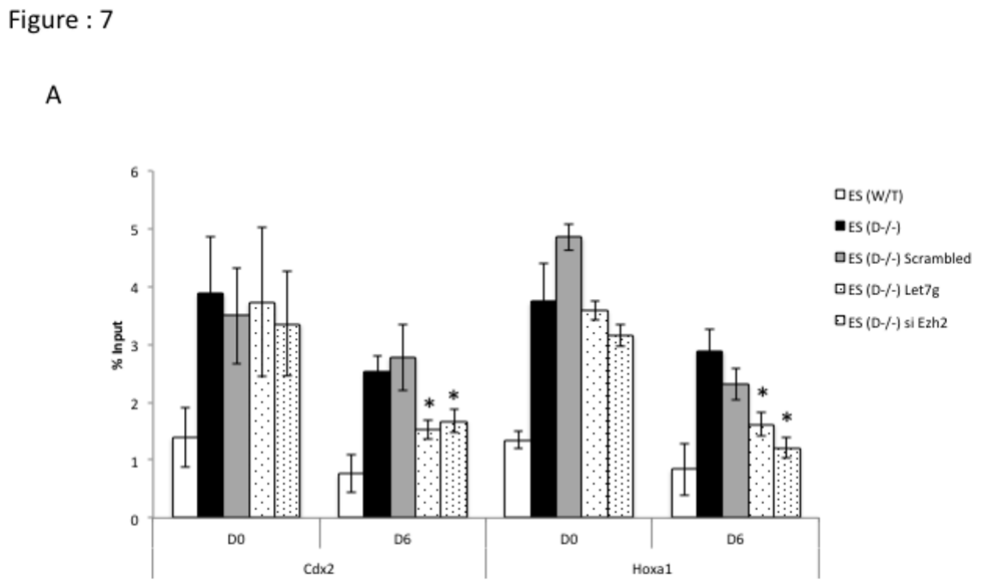
Transfecting Dicer^-/-^ ES cells with let-7g miRNA and siRNA targeting *Ezh2* reduces H3K27me3 at *Hoxa1* and *Cdx2* during retinoic acid induced differentiation. Cells were treated with let-7g miRNA and siRNA-targeting *Ezh2* along with a scrambled negative control as described in the methods section and chromatin immunoprecipitated using an H3K27me3 specific antibody. Thereafter enrichment of H3K27me3 at *Hoxa1* and *Cdx2* loci were assayed by quantitative real time PCR. * Indicates that the differences at a given time point between Dicer^-/-^ ES cells and Dicer^-/-^ ES cells treated with si*Ezh2, or* let-7g mimic were significant at 0.05 confidence levels when a students t-test was performed.

To functionally validate our hypothesis we overexpressed mature let-7g in Dicer^-/-^ ES cells and assayed the expression levels of developmentally significant genes, which are critical in determining cell fate. Upon over-expression of mature let-7g, both *Lin28*b and *Ezh2* transcript levels were down-regulated in Dicer^-/-^ ES cells (Figure 5A and 5B), while *Cdx2* and *Hoxa1* transcript levels were increased (Figure 5A). In sharp contrast, overexpression of let-7g has very little effect on pluripotency genes (Figure S2: A). In order to assess the effect of *Ezh2* by itself on the expression levels of genes in the absence of concomitant down-regulation of *Lin28* with *Ezh2* we used siRNAs targeting *Ezh2*. We found that siRNAs to *Ezh2* were sufficient to increase levels of *Cdx2* and *Hoxa1* transcripts in Dicer^-/-^ ES cells (Figure 6A). However, siRNAs targeting *Ezh2* had no significant effect on *Lin28b, Gcnf* and the pluripotency genes (Figure 6B and Figure S3: A) To test the impact of let-7g and siRNAs to *Ezh2* on patterns of H3K27me3 at *Hoxa1* and *Cdx2* loci in Dicer^-/-^ ES cells we carried out treatments with let-7g and *siEzh2* along with respective controls and chromatin immunoprecipitated genomic DNA from the cell lines using an H3K27me3 specific antibody. A significant reductions of H3K27me3 was found at *Hoxa1* and *Cdx2* in Dicer^-/-^ ES cells compared to mock and control treated samples (Figure 7A).

## Discussion

The role of *Dicer*, in posttranscriptional regulation of embryonic stem cell genes is well established. Sinkkonen and co-workers have shown that regulating DNA methylation in ES cells require the miR-290-295 cluster which facilitates appropriate DNA methylation at developmentally critical genes by downregulating *Rbl* a known repressor of the enzymes DNA methyltransferases 3A and 3B in ES cells [12]. Here we report that *Dicer*-dependent histone methyl modifications can affect transcription of key developmental genes by functioning as an additional layer of epigenetic regulation in conjunction with DNA methylation. To examine the role of *Dicer* in regulating histone modifications that are critical to self-renewal and differentiation of ES cells we first examined genome-wide changes in H3K9me2 in the presence and absence of *Dicer* and subsequently measured local changes in H3K27me3 and H3K4me3 in the promoters of key regulators of self-renewal and pluripotency (*Oct4*, *Nanog*, *Sox2* and *Ronin*), differentiation (*Cdx2* and *Hoxa1*) and genes promoting the transition between these states (*Lin28*b and *Gcnf*). The critical role, which *Dicer* plays in mammalian embryogenesis, is highlighted by the fact that mouse embryos lacking *Dicer* do not successfully progress beyond day 6.5 in development [8,9]. Using the ability to maintain *Dicer*-deficient ES cells in long-term culture and RA-mediated differentiation cues to our advantage we specifically show that loss of *Dicer* can lead to changes in the landscape of the embryonic stem cell epigenome by impacting histone modifications in genes critical for embryonic stem cell differentiation.

Loss of *Dicer* in particular led to a global increase in H3K9me2 modifications at over 900 CpG islands. In order to examine how H3K9me2 modifications work with other histone modifications at the promoters of genes critical for stem cell function we also integrated data for H3K4me3 and H3K27me3 modifications. Upon carefully evaluating genes regulating different aspects of ES cell functions (pluripotency, differentiation and transition between the two states) we uncovered a higher level of complexity where the presence and comparative abundance of transcriptionally favorable (H3K4me3) marks versus transcriptionally unfavorable (H3K27me3) marks affect mRNA expression levels. We further found that maintaining this intricate balance is *Dicer*-dependent. An increase in H3K27me3 in the promoters of differentiation genes *Hoxa1* and *Cdx2* in Dicer^-/-^ES cells was coincident with an inability to up-regulate these genes at the same rate as WT ES cells upon RA-induced differentiation. siRNAs and microRNA let-7g rescued this effect by down-regulating *Ezh2* suggesting that *Ezh2* up-regulation is in part responsible for increased H3K27me3 and decreased rates of up-regulation of differentiation genes in Dicer^-/-^ES cells. The presence or absence of Dicer had no impact on H3K4me3/H3K27me3, transcript levels or rate of down-regulation of pluripotency genes Oct4, Nanog and Sox2. By contrast, the promoter of *Lin28b* regulating the transition from self-renewal to differentiation was associated with a significant increase H3K4me3 in Dicer^-/-^ES coincident with down-regulation of *Lin28b* at a lower rate. *Lin28*b mediated repression of let-7g, has been shown to block ES cells from differentiating [23]. *Gcnf*, established to be critical for the repression of *Oct4* and *Nanog* [22,25,26] was found to be up-regulated at a higher rate in Dicer^-/-^ES cells.

**Figure 8:**
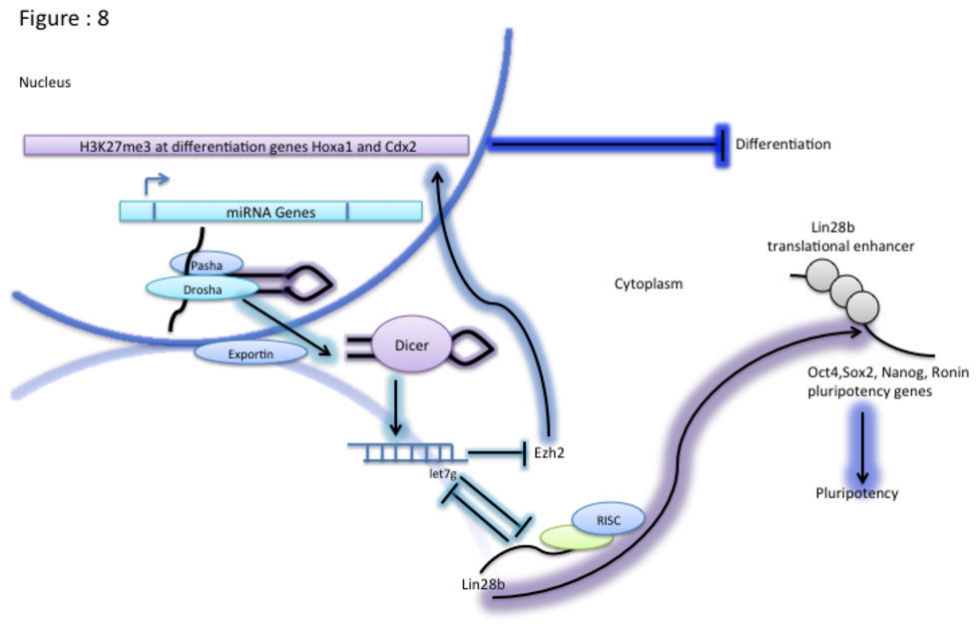
Putative model explaining *Dicer* mediated regulation of mES cell differentiation. When ES cells are induced to differentiate by RA treatment let-7g levels increase. Hence let-7g suppresses *Ezh2* resulting in reduced H3K27me3 favoring transcription of *Hoxa1* and *Cdx2* differentiation genes. As let-7g increases during differentiation *Lin28b* levels are reduced. As a result translational enhancement of pluripotency factors by *Lin28b* is reduced, this event in turn favors differentiation.

A putative model to explain our findings is shown in Figure 8. It is already known that the transition of ES cells from the stem cell state to the differentiated state in response to RA requires the down-regulation of *Lin28b* and the subsequent differentiation of ES cells require the upregulation of differentiation genes such as *Hoxa1* and *Cdx2*. Our data suggest that Dicer plays a critical role in balancing the transcriptionally favorable and unfavorable histone modifications at these genes that regulate RA-induced differentiation of ES cells. We propose that one of the major impacts of loss of Dicer is the disruption of let-7g mediated post-transcriptional repression of *Ezh2* during RA-induced differentiation. This in turn results in a cascade of events whereby increased H3K27me3, leads to the attenuated up-regulation of genes critical for differentiation such as *Hoxa1* and *Cdx2* in Dicer^-/-^ES. In addition genes regulating the transition from stem cell state to the differentiated state are also perturbed in their response to RA-induction. *Lin28b*, which is typically down-regulated upon RA-induced ES cell differentiation, is down-regulated at a lower rate in Dicer^-/-^ES cells. The 3’ UTR based repression of *Ezh2* by let-7g mimics introduced into Dicer^-/-^ES cells is sufficient to rescue the increase in H3K27me3 and the resulting attenuation in up-regulation of *Hoxa1* and *Cdx2*. In addition, the rapid increase in levels of let-7g levels upon RA induction leads to a decrease in levels of *Lin28b* via 3’ UTR based repression in WT ES cells. Since *Lin28b* is a translational enhancer of pluripotency factors the ES cells are now free to differentiate. Collectively, the attenuated up-regulation of genes critical for differentiation such as *Hoxa1* and *Cdx2* and the attenuated down-regulation of *Lin28b* render *Dicer* deficient ES cells unable to differentiate upon retinoic acid induction.

Understanding the interactions between transcription factors, miRNAs and the epigenetic landscape is a fundamental requirement to develop strategies aimed at cellular reprogramming. Our observations suggest that a single miRNA (let-7g) alone can significantly alter epigenetic histone modifications at genes critical for maintenance of the stem cell state and their ability to differentiate when exposed to the appropriate cues. Our findings suggest that *Dicer* mediated pathways involving miRNAs, PcGs and epigenetic marks, could be potentially manipulated to direct cellular fate in engineering cells for therapeutic applications in future.

## Materials and Methods

### Cell Culture

mES cells were cultured on gelatin coated plates using DMEM (Gibco, Invitrogen, Carlsbad California catalog number-31053) containing 15% FBS (Invitrogen, Carlsbad California catalog number-10439-024), 1X Penstrep (Gibco, Grand Island New York catalog number-15140-122), 1X Glutamine (Gibco, Grand Island New York catalog number-25030-024), 1X non-essential amino acids (Gibco, Grand Island New York catalog number-11140-035), 1X Pyruvate Solution (Gibco, Grand Island New York catalog number- cat#11360), 0.1mM 2-mercaptoethanol (Sigma, St. Louis Missouri catalog number-M7522) and 1000U/ml LIF (LIF 2010, Millipore, Billerica, Massachusetts) during day 0 by seeding at a density of 250,000 cells per well. LIF containing media was replaced with RA containing media to induce differentiation after day 0. Thereafter the cells were maintained in RA containing medium by feeding on a daily basis. Mouse WT ES and Dicer^-/-^ ES cells were a generous gift from Gregory Hannon, Cold Spring Harbor Laboratories [10].

### miRNA and siRNA transfections

Mature let-7g miRNAs and *Ezh2* siRNAs were purchased from Invitrogen Inc, Carlsbad, USA. Cells were transfected with let-7g and si*Ezh2* RNAs on day 0 on six well format plates using standard protocols for Lipofectamine 2000 transfection reagent (Catalog number11668-019, Invitrogen, Carlsbad, California). Each well was transfected with 5 µl of Lipofectamine 2000 reagent and let-7g miRNA at a final concentration of 10 nM and a final concentration of 50 nM si*Ezh2*. In order to sustain overexpression the cells were re-transfected using the same protocols at day 3 after RA induction.

### RNA Extraction

RNA was extracted from the cell lines using a Qiagen miRNAeasy mini extraction kit (Catalog number 217004), Qiagen, Maryland, USA) according to manufactures instructions. In order to make sure that the RNA samples were free of DNA contamination an on column DNAse treatment was carried out according to manufactures’ protocols using a Qiagen DNAse reagent kit.

### Quantitative real time PCR

Quantitative real-time reactions were performed on Chromatin immunoprecipitated DNA and reverse transcribed RNA samples on a 96 well format Applied Biosystems 7500 real time PCR machine using SYBR green dye according to manufacturers protocols (catalog number 4385112, Applied Biosystems, New Jersey, USA). Mouse 18S ribosomal subunit gene mRNA was used as an endogenous control. mRNA fold changes of candidate genes were calculated by normalizing against the ES day zero samples in respective experiments of a given panel. Total mRNA was extracted from cell samples using a Qiagen RNA easy kit (catalog number 74104, Qiagen, California, USA). In order to ensure the RNA samples were free of genomic contamination an on column DNAse digestion treatment was carried out using a Qiagen RNase free DNase kit (catalog number 79254, Qiagen, California, USA). CDNA synthesis of isolated RNA was carried out using a Taqman Reverse transcription kit according to manufacturers protocol (catalog number N8080234, Applied Biosystems, New Jersey, USA).

### Chromatin Immunoprecipitation

Chromatin immunoprecipitation was performed on genomic DNA samples from cell lines using antibodies specific to H3K4me3, HK9me2 and H3K27me3 as previously described [27]. 5x10^6^ cells were used for each ChIP assay. The cells were Formaldehyde (1%) cross-linked for 10 minutes. The cell lysates were sonicated to shear DNA to lengths between 200 and 1000 bp. The following antibodies were used for the chromatin immunoprecipitation assays: Rabbit anti-Dimethyl-H3K9 (Cat# 39765, Active Motif 914 Palomar Oaks Way # 150, Carlsbad, CA) 92008-6509Rabbit anti-Trimethyl- H3K4 (Cat# 07-473, Upstate Biotechnology ,inc., Lake Placid, NY), Rabbit anti-Trimethyl-H3K27 (Cat# 07-449, Upstate Biotechnology ,inc., Lake Placid, NY ). The DNA/antibody complexes were pulled down by the protein A/G plus-agarose (cat# sc- 2003, Santa Cruz Biotechnology, Inc., Santa Cruz, CA) Enrichment of a given histone modification in the promoter regions of respective genes were calculated as a percentage of the input samples used for each experiment.

### Solexa Library preparation

Libraries for ChIP-Seq analysis were prepared using standard protocols of the Solexa Illumina paltform provided by the manufacturers’ as well as our previous descriptions [27].

### Genomic Mapping

Genomic mapping of chromatin immunoprecipitated sequence libraries for ES and Dicer^-/-^ were performed using the PASH algorithm as previously described [20]. Uniquely mapping reads were selected, then read coverage was computed over 100bp windows tiling across the entire genome. The read density maps were normalized employing a quantile normalization step. For each genomic window the difference between ES and Dicer^-/-^ and the associated p-values were computed; windows with a false discovery rate (FDR) below 0.001 were selected, and then a segmentation algorithm was applied. Finally, genomic feature sets such as gene promoters, gene exons, microRNAs, piRNA clusters, CpG islands and others were annotated for individual elements with enriched bindings in either ES or Dicer^-/-^. The virtual genome map in Figure 1A was generated using the VGP feature of the Genboree tool (http://www.genboree.org/java-bin/login.jsp).

### Primers

All primers for real time quantitative PCR and ChIP-qPCR were designed using the publicly available “Primer 3” tool (http://frodo.wi.mit.edu/primer3/). Primers for qRT-PCRs as listed in table **S1** were designed to span intronic regions whenever possible to avoid background resulting from genomic contamination of RNA samples. Primers for ChIP-qPCR were designed to span promoter regions of the assayed genes derived from the Cold Spring Harbor mammalian promoter database (http://rulai.cshl.edu/CSHLmpd2/). The complete lists of primers used for ChIP-qPCR assays are listed in table **S2** **.**

### Statistical analysis

Statistical analyses were carried out using Graph pad Prism and Excel software. Students’ t-tests were carried out to assess significance in differences in quantitative real time PCR results while false differential ratios were calculated to assure quality of chromatin immunoprecipitated sequence mapping. Unless otherwise stated all statistical analyses were carried out and tested at confidence levels of 0.05 or less.

## Supporting Information

Figure S1 (A, B, C, D, E, F and G) ChIP-qPCR results showing H3K9me2 enrichment in Oct4, Sox2*, Nanog, Lin28b, Gcnf*, Cdx2 and *Hoxa1* promoters respectively.
(TIFF)

Figure S2 (A) Pluripotency factors *Oct4*, *Sox2* and *Nanog* remain relatively unchanged upon transfecting Dicer^-/-^ ES cells with let-7g.
(TIFF)

Figure S3 (A) Transfecting Dicer^-/-^ ES cells with siRNA targeting *Ezh2* has no significant effect on pluripotency gene mRNA.
(TIFF)

Table S1 List of primers used for qRT-PCR assays.
(DOC)

Table S2 List of primers used for ChIP-qPCR assays.
(DOC)

Table S3 **Genomic Loci of H3K9me2 in ES and Dicer-/- ES cells.**
(XLS)
