# Supplementary material for: Chromatin Changes in Dicer-Deficient Mouse Embryonic Stem Cells in Response to Retinoic Acid Induced Differentiation
Source: PLoS One. 2013 Sep 9;8(9):e74556. doi: 10.1371/journal.pone.0074556 (PMC3767645; doi:10.1371/journal.pone.0074556)
Supplement: Table S1 — List of primers used for qRT-PCR assays. (DOC) [file pone.0074556.s004.doc]

**Table S1: List of primers used for qRT-PCR assays.**

| **Gene** | **Forward primer** | **Reverse primer** |
| --- | --- | --- |
| **Oct4** | AGAGGGAACCTCCTCTGAGC | CCAAGGTGATCCTCTTCTGC |
| **Sox2** | CACAACTCGGAGATCAGCAA | CGGGGCCGGTATTTATAATC |
| **Nanog** | ATGCCTGCAGTTTTTCATCC | GAGCTTTTGTTTGGGACTGG |
| **Ronin** | AAGAGGGCTTCCCTGACACT | CGGATGCTACCCTTCATCTC |
| **Lin28b** | GAGAGGGAAATCCCTTGGATA | TGTTACCCGTATTGACTCAAGG |
| **Gcnf** | TTGCAACAAACGGGTGTATC | GGCATGCCATCTTCTCTGAT |
| **Hoxa1** | GCAGACCTTTGACTGGATGA | AGCTCTGTGAGCTGCTTGGT |
| **Cdx2** | ACCTGTGCGAGTGGATGC | TGAAACTCCTTCTCCAGCTC |
| **Ezh2** | GCGGAAGCGTGTAAAATCAG | GCTGCTTCCACTCTTGGTTT |
| **Setd2** | CCGAAGATGGGGGATTTCTA | TTTTGTCTTCGTGCCTTTGG |
| **G9a** | CCTGAGCTTCGGAACAAAGA | ATTGACACAGGGGATGGGTA |
| **Ash1l** | CCAGTTGCCGGAAGAAATTA | TGATCGCTGGGAAGGTTTAT |
| **18S** | AGTTCCAGCACATTTTGCGAG | TCATCCTCCGTGAGTTCTCCA |
