# Supplementary material for: Chromatin Changes in Dicer-Deficient Mouse Embryonic Stem Cells in Response to Retinoic Acid Induced Differentiation
Source: PLoS One. 2013 Sep 9;8(9):e74556. doi: 10.1371/journal.pone.0074556 (PMC3767645; doi:10.1371/journal.pone.0074556)
Supplement: Table S2 — List of primers used for ChIP-qPCR assays. (DOC) [file pone.0074556.s005.doc]

**Table S2 : List of primers used for ChIP-qPCR assays.**

| **Gene** | **Forward primer** | **Reverse primer** |
| --- | --- | --- |
| **Oct4** | TGGGCTGAAATACTGGGTTC | TTGAATGTTCGTGTGCCAAT |
| **Sox2** | GCGGCCCCTGCATCC | TTCAGCTCCGTCTCCATCAT |
| **Nanog** | TTTGGTTGTTGCCTAAAACCTT | CTGCAGGCATTGATGAGG |
| **Ronin** | CGTCGAAGAGCTCGAGAGG | CGTCCTTGGGAAACGTGTAG |
| **Lin28b** | ATCAAGATGTTAGACTGATGCTGAA | GAGAAAAGAAGAGAGGAATCACAG |
| **Gcnf** | CCCGTGGAAGACCAGGAC | GCTAGGTGGCCGTTCGTC |
| **Hoxa1** | CATATCATTTTTCTTCTCTGGTCCT | ACTGCCAAGGATGGGGTATT |
| **Cdx2** | CCTCTGGCAGCCTTCAAC | CGCACGGAGCTAGGATACAT |
